# Environmental Coupling of Selection and Heritability Limits Evolution

**DOI:** 10.1371/journal.pbio.0040216

**Published:** 2006-06-13

**Authors:** A. J Wilson, J. M Pemberton, J. G Pilkington, D. W Coltman, D. V Mifsud, T. H Clutton-Brock, L. E. B Kruuk

**Affiliations:** **1**Institute of Evolutionary Biology, University of Edinburgh, Edinburgh, United Kingdom; **2**Department of Biological Sciences, University of Alberta, Edmonton, Canada; **3**Department of Zoology, University of Cambridge, Cambridge, United Kingdom; University of North CarolinaUnited States of America

## Abstract

There has recently been great interest in applying theoretical quantitative genetic models to empirical studies of evolution in wild populations. However, while classical models assume environmental constancy, most natural populations exist in variable environments. Here, we applied a novel analytical technique to a long-term study of birthweight in wild sheep and examined, for the first time, how variation in environmental quality simultaneously influences the strength of natural selection and the genetic basis of trait variability. In addition to demonstrating that selection and genetic variance vary dramatically across environments, our results show that environmental heterogeneity induces a negative correlation between these two parameters. Harsh environmental conditions were associated with strong selection for increased birthweight but low genetic variance, and vice versa. Consequently, the potential for microevolution in this population is constrained by either a lack of heritable variation (in poor environments) or by a reduced strength of selection (in good environments). More generally, environmental dependence of this nature may act to limit rates of evolution, maintain genetic variance, and favour phenotypic stasis in many natural systems. Assumptions of environmental constancy are likely to be violated in natural systems, and failure to acknowledge this may generate highly misleading expectations for phenotypic microevolution.

## Abstract 2

An analysis of birth weight in Soay sheep reveals that environmental heterogeneity can constrain evolution and suggests that rates of evolutionary change may be lower in natural populations than expected from theory.

## Introduction

Evolution is expected to occur when selection acts on a trait that has a heritable basis of phenotypic variation. Quantitative genetic models allow an evolutionary trajectory to be predicted from the strength of selection and the amount of genetic variance, usually expressed as the heritability, *h* ^2^ [ 1]. However, while simple theoretical models assume a constant environment, environmental heterogeneity has long been recognised as an important factor influencing the evolutionary dynamics of fitness-related traits in the wild [ 2]. Specifically, selection can vary considerably from year to year within a population [ 3, 4], and it is increasingly recognised that environmental conditions also influence the heritability on which any response to selection depends [ 5, 6]. Although these observations generate an expectation of an environment-driven coupling of the magnitude of selection and heritability, to our knowledge, no prior study has combined estimates of trait heritability with estimates of the strength of selection across a range of environmental conditions in order to fully assess the evolutionary implications of environmental heterogeneity.

Here we examine the simultaneous effects of environmental variation on selection and heritability, using data from a long-term study of Soay sheep *(Ovis aries)* on the Scottish island of Hirta, St. Kilda [ 7]. This system is ideal for our purposes, because of the availability of a large volume of multigenerational data (including both phenotypic and pedigree data), which covers a 20-y period (1985–2005), characterised by extensive variation in environmental quality. In particular, the population has been subject to repeated episodes of very high mortality that occur primarily in response to density and climatic conditions [ 8, 9]. By studying a single population in which the major axis of environmental variation is temporal, we can minimize the potential to confuse environmental effects on genetic variance with demographic effects that may arise with comparisons between distinct populations over a spatially heterogeneous environment. Our approach also contrasts with previous empirical investigations of the impact of environmental quality on genetic variances, which have relied on comparison between environments defined as either good or bad [ 10]. While such a dichotomous categorisation may be useful under some circumstances, it may be rather arbitrary where environmental quality shows a more continuous range.

Here, we employ the analytical technique of “random regression” to model genetic variance as a function of a continuously varying environment. This not only allows a more realistic model of environmental variation, but can also provide statistical benefits [ 11]. In particular, random regression allows more efficient use of data by avoiding subdivision of records into environment-specific traits with reduced sample sizes. This is particularly useful for analyses of natural populations where sampling constraints normally limit data availability, and hence statistical power, by comparison with artificial systems [ 12]. To date, random-regression models have been used primarily to model trait ontogenies in commercial livestock populations, with genetic effects modelled as covariance functions of age [ 13, 14]. Though novel in an evolutionary context, their application to testing of genotype-by-environment interaction does have precedent in the animal-breeding literature [ 14].

We focused on the trait of lamb birthweight (denoted in Equation 1 by *BWT*) and its association with juvenile mortality in the Soay sheep population. Of the total mortality of sheep, 24% occurs during the neonatal period (from birth in April or May until October 1 of the same year), making it a critical episode for viability selection [ 15]. Early body-size traits are under strong positive directional selection (i.e., larger individuals having higher fitness), with differential juvenile mortality resulting in significant selection through lifetime fitness [ 4, 16, 17]. While birthweight has been shown to be positively associated with neonatal survival [ 15], previous analyses have found no evidence for non-linear (i.e., stabilising or disruptive) selection on this trait [ 17].

Birthweight is also heritable, as a consequence of strong maternal genetic effects, even though additive genetic variance ( *V*_A_) is relatively low [ 18] (see Table 1 for a glossary of quantitative genetic parameters and their abbreviations used herein). Maternal genetic effects occur when the genotype of a mother influences the offspring's phenotype (independently of the direct effect of the genes it inherits from her), and may often represent an important source of heritable phenotypic variation [ 19]. Although only a limited number of studies have directly estimated maternal genetic effects in natural populations ([ 12, 20]), they have previously been shown to comprise the major heritable component of phenotypic variance for birthweight in Soay sheep [ 18].

**Table 1:**
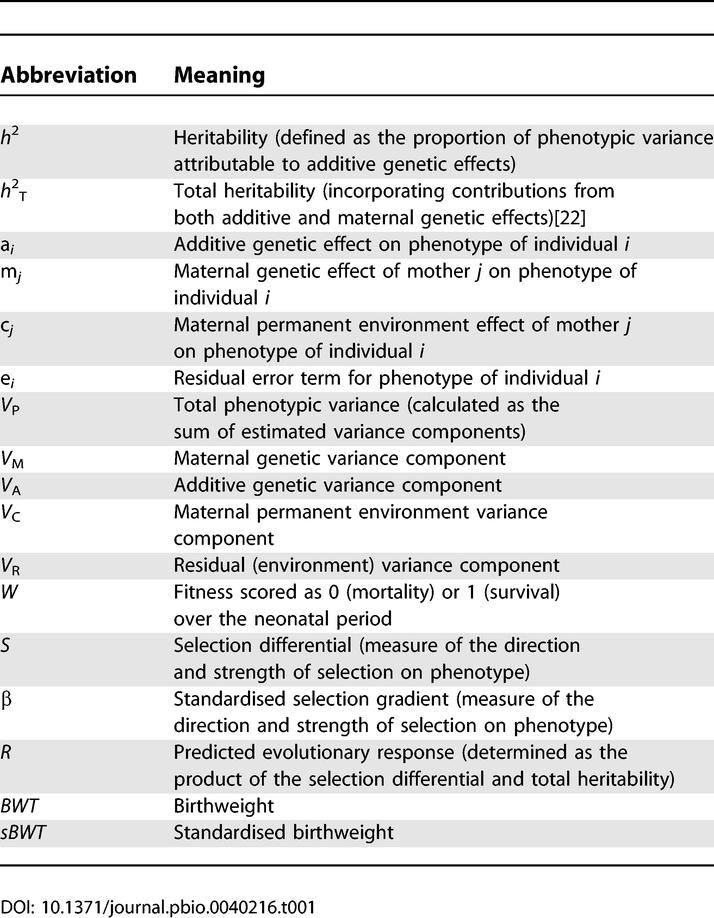
Glossary of Terms and Abbreviations Used in Quantitative Genetic Models

Despite both positive directional selection and heritable variation [ 17, 18], annual mean birthweight has not increased over the study period and actually shows a non-significant declining trend (linear regression on time; slope = −0.016 kg/y, *p* = 0.15, *n* = 19). We therefore tested the hypothesis that environmental heterogeneity limits phenotypic evolution through effects on heritability or selection, or both simultaneously. To do this, we first examined the impact of environmental variation on the heritable basis of birthweight by modelling levels of genetic variance across a variable environment. Secondly, we tested for systematic variation between years of differing environmental conditions in the strength of selection acting over the neonatal period.

## Results/Discussion

### Heritable Variation in a Variable Environment

We estimated heritable variation in Soay sheep birthweight using an animal model approach [ 21], which allows the observed phenotypic variance to be separated into genetic and environmental components. Female sheep are long-lived (with a maximum recorded longevity of 16 y on Hirta), so that individual mothers produce offspring in years of differing environmental conditions. Consequently, we were able to use a “random-regression animal model” [ 14] in which the main source of heritable variation, the maternal genetic effect, was explicitly modelled as a function of environmental quality.

To model genetic variation for birthweight, we defined a measure of environmental quality *(E)* based on the level of neonatal mortality in each year (see Materials and Methods). An environment was defined as being of intrinsically poor quality if lamb survival was low, and of good quality if survival was high. We then fitted the maternal genetic effect as a polynomial function of *E* in the random-regression animal model. By changing the order of the polynomial function used, we compared successively more complex models of the maternal genetic effect. If a zero-order function (i.e., a constant) is used, then an individual's maternal genetic effect cannot change with *E* and, consequently, variance in these effects (i.e., the maternal genetic variance) is also constant across environments. In contrast, fitting higher-order polynomial functions allows systematic variation in the amount of maternal genetic variance across environments to be explicitly tested for.

**Figure 1:**
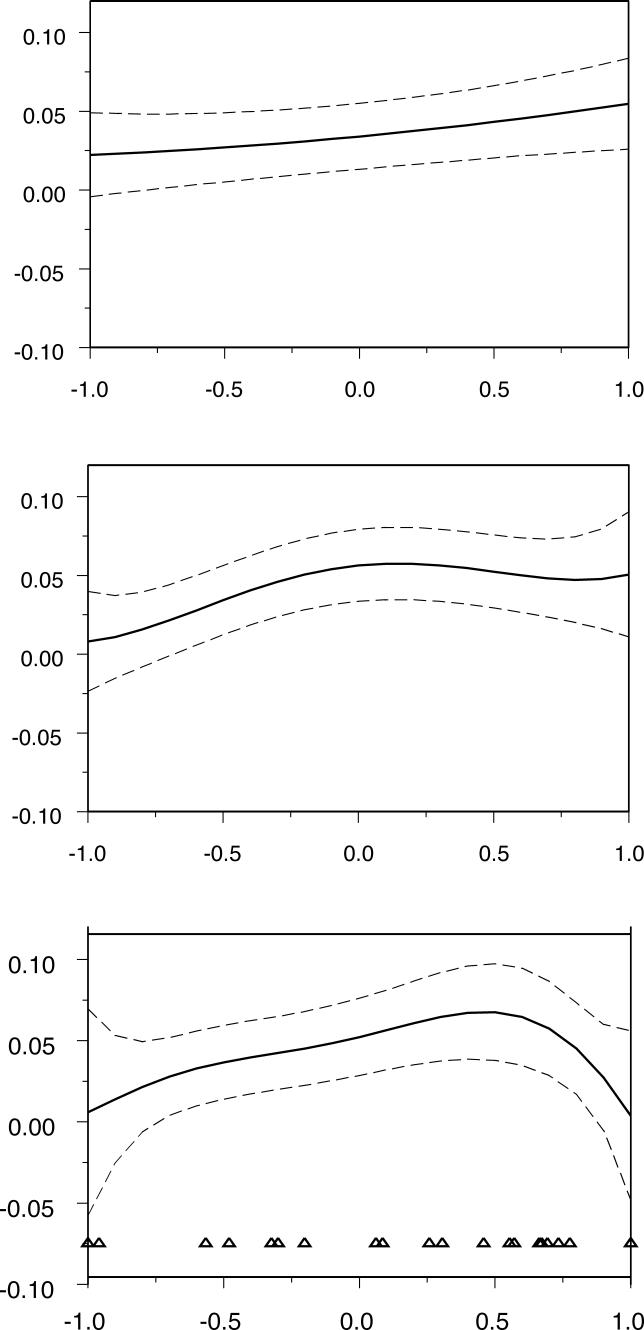
Estimated Maternal Genetic Variance across Environments Dashed lines indicate approximate 95% confidence limits to the estimated maternal genetic variance ( *V*_M_), while triangles on the lower panel indicate the actual distribution of environmental quality *(E)* across years. All three models suggest a general increase in *V*_M_ as environmental quality increases.

We fitted random-regression animal models with the maternal genetic effect modelled using zero, first, second, and third-order functions of environmental quality (denoted as Models I, II, III, and IV, respectively, in Table 2). Statistically, the best model fitted was with a third-order function (Model IV) under which the maternal genetic variance ( *V*_M_) showed a general increase with *E* ( Table 2; Figure 1). Under Model IV, the additive, maternal permanent environment plus residual components of variance (with standard errors) were estimated as *V*_A_ = 0.020 (0.009), *V*_C_ = 0.015 (0.008), and *V*_R_ = 0.116 (0.008), respectively. As a consequence of increasing *V*_M_, the evolutionary potential of birthweight, as indicated by the total heritability *h* ^2^_T_ (a measure that includes contributions from both the additive and maternal genetic effects [ 22]), also increased with environmental quality. This pattern of increased heritability in good conditions is consistent with the emerging trend from empirical studies of wild vertebrate populations [ 10, 23]. Given the observed distribution of *E* across years ( Figure 1), it is apparent that the decline in *V*_M_ at the uppermost values of *E* is largely driven by data from a single year (1987). Analysis with 1987 data excluded confirmed a strong trend of increasing *V*_M_ with *E,* as did simpler models where we used first- or second-order functions of environmental quality (Models II and III; Figure 1). All models in which *V*_M_ could vary with environment were statistically superior to a conventional animal model with a constant maternal genetic effect (Model 1 in Table 2).

**Table 2:**
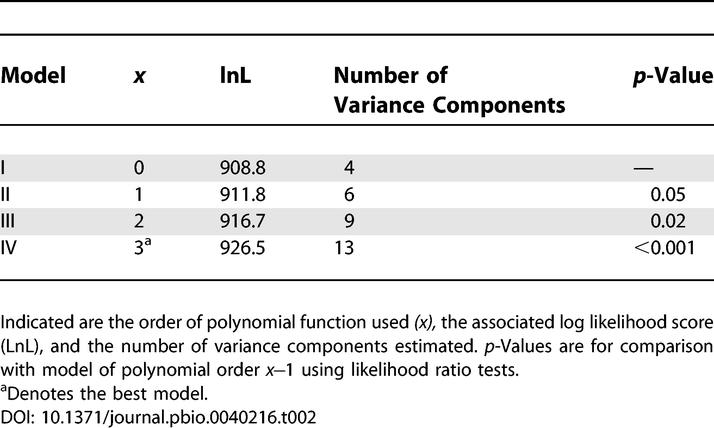
Comparison of Random-Regression Animal Models Fitted

**Figure 2:**
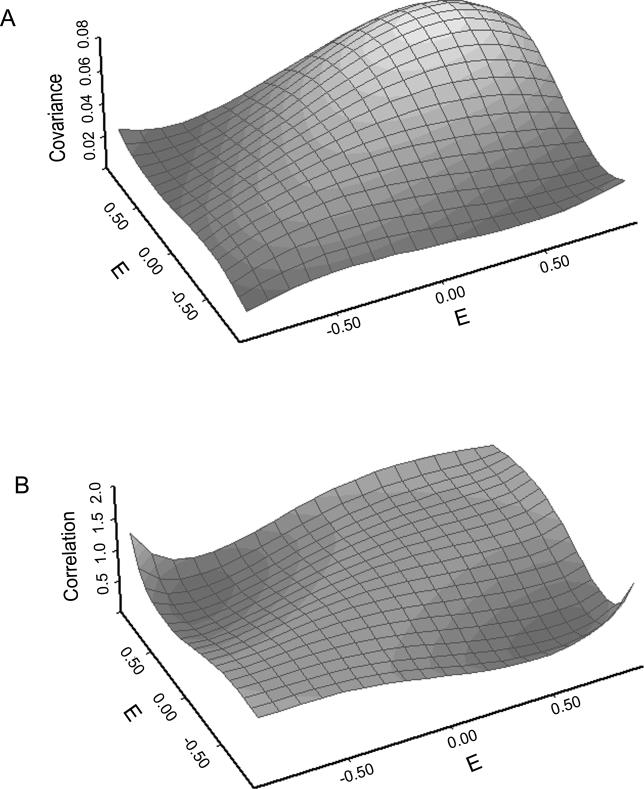
Maternal Genetic Covariance and Maternal Genetic Correlation Surfaces for Birthweight Expressed across Environments (A) Maternal genetic covariance; (B) maternal genetic correlation. The maternal genetic correlation *r _M_* is close to +1 over most of the surface, suggesting that the same maternal loci are involved and have qualitatively similar effects on offspring birthweight across all environments.

Maternal genetic covariances between environments were also estimated under Model IV, and were found to be uniformly positive ( Figure 2). Rescaling the covariances to a correlation scale showed that maternal genetic correlations were close to +1 over most of the surface ( Figure 2). The strength of these correlations indicates that variation in maternal performance likely involves the same loci in all environments, while the positive sign shows that qualitative effects of allelic variants are conserved as *E* changes. Nevertheless, maternal genotype-by-environment interaction is shown by the environmental dependence of *V*_M_.

### Selection in a Variable Environment

Our finding of environmental variation in the heritable variance for birthweight was complemented by similar systematic variation in strength of selection. The relationship between phenotype and fitness (defined as neonatal survival) was investigated using generalised linear mixed modelling and standard regression-based methods [ 24], and confirmed prior expectations of positive directional selection on birthweight. Thus, generalised linear mixed modelling shows fitness increases with standardised birthweight (denoted in Equation 3 by *sBWT*), as well as with environmental quality *E* ( Table 3). However, of more interest is the relationship between the selection regime and the quality of the environment. The significant negative interaction between standardised birthweight and *E* indicates that the strength of the positive relationship between fitness and phenotype is reduced in better environments. Annual estimates of selection differential *S* were consistently positive, but ranged widely from +0.025 to +0.319 kg across years. Corresponding estimates of the standardised selection gradient β (with standard errors) are useful for comparison with other studies, and ranged from +0.038 (± 0.019) to a maximum of +0.529 (± 0.064). This confirms that, in some years, directional selection was considerably stronger than the median value of 0.16 reported in the literature [ 25].

**Table 3:**
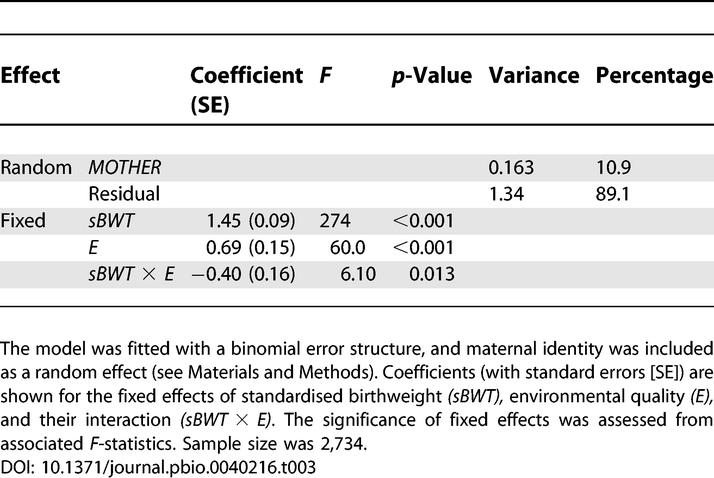
Mixed-Model Analysis of Offspring Fitness

**Figure 3:**
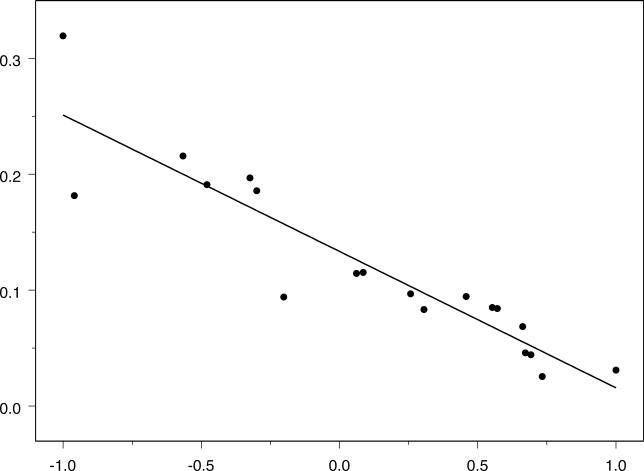
Selection Differential on Birthweight against Environmental Quality Each point represents the values measured in 1 y, and the solid line shows the least-squares linear regression of selection differential *(S)* on environmental quality *(E).* The strength of selection acting on birthweight declines as the environmental quality improves.

Annual selection differentials were also strongly negatively correlated with environmental quality (Pearson's correlation *r* = −0.919, df = 17, *p* < 0.001), confirming that positive directional selection on birthweight is weaker in good environments ( Figure 3). Pooling across years, the overall selection differential *S* was estimated at +0.138 kg. This is slightly greater than the mean of the annual estimates (+0.120 kg), suggesting a slight upward bias in the overall selection estimate through environmentally induced positive covariance between phenotype and fitness [ 26].

**Figure 4:**
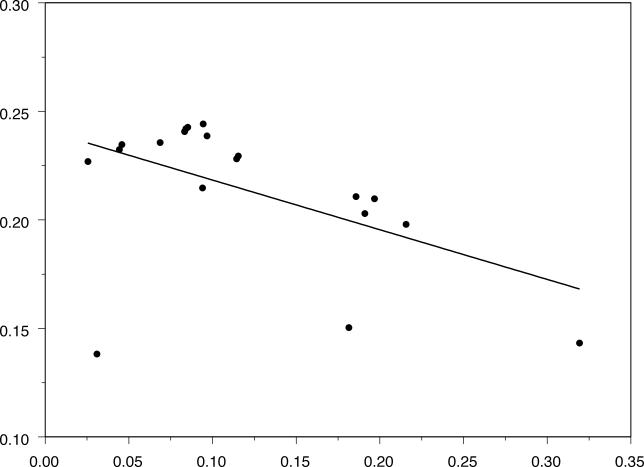
Selection Differential on Birthweight *(S)* against Estimated Total Heritability ( *h* ^2^_T_) Each point represents the values measured in 1 y. The solid line shows the least-squares linear regression of estimated total heritability ( *h* ^2^_T_) on selection differential *(S).* The strength and direction of this relationship is unchanged by removal of outliers in the lower portion of the graph.

Thus, maternal genetic variance for birthweight increases with environmental quality, while the strength of selection decreases. This shared dependence on environmental quality results in a negative correlation between the strength of selection on birthweight and the amount of heritable variation. Accordingly, annual estimates of the total heritability ( *h* ^2^_T_), obtained under Model IV, were strongly negatively correlated with annual selection differentials *S* ( Figure 4; Pearson's correlation *r* = −0.512, df = 17, *p* = 0.025).

### Conclusions

Evolution of increased birthweight in response to differential neonatal mortality is therefore limited by either a lack of heritable variation in poor environments or by a reduced strength of selection in good environments. Annual phenotypic responses *(R)* to selection through neonatal mortality, determined using the breeder's equation [ 1] as the product of *h* ^2^_T_ and *S,* were small but showed a 10-fold range in magnitude (from 0.004 to 0.046 kg per generation). The weighted geometric mean response (see Materials and Methods) was 0.019 kg per generation (or approximately 0.008 kg per year, based on an estimated generation time of 2.4 y). This is less than the predicted response if environmental heterogeneity is not considered ( *R* = 0.024 kg per generation, with selection estimated across all years and total heritability estimated from a conventional animal model). Predicted responses should be interpreted with some caution since many assumptions of the breeder's equation will likely be violated in this case. For example, these predictions reflect selection through neonatal mortality only, while birthweight may co-vary with multiple fitness components [ 17] and with other traits under selection. Additionally, the relative importance of different selective mechanisms may vary with environmental conditions, such that more sophisticated approaches to estimating selection will be needed to effectively predict phenotypic change [ 27, 28]. Nevertheless, our results clearly demonstrate the potential for environmental heterogeneity to significantly affect estimates of evolutionary parameters in natural populations [ 6, 26, 29].

Our findings thus highlight the differences between populations in natural environments and those in the controlled, constant environments for which quantitative genetic theory has been developed. While suitable long-term data will not always be available, increased efforts should be made, wherever possible, to fully consider the implications of environmental heterogeneity for both selection and the genetic variation on which it acts. In particular, if favourable environmental conditions are generally associated with low levels of selection but with high levels of heritability, and vice versa, then rates of evolutionary change may be much lower than those expected from values averaged across environments, providing an explanation for the frequently-observed stasis [ 29].

## Materials and Methods

### Study system.

Soay sheep were introduced to the Scottish archipelago of St. Kilda in the Bronze Age, and to the main island of Hirta (lat 57°49′N, long 08°34′W) in 1932 shortly after the human evacuation of St. Kilda. Data relating to birth, death, reproduction, and phenotype have been collected for individually tagged animals resident in the Village Bay area since 1985 [ 7]. Lambs are generally captured within a few days of birth, and we define an individual's birthweight as the residual from a linear regression of capture weight on age [ 17]. The pedigree contains 6,117 individual records, with 3,355 maternal links and 1,615 paternal links (from 784 distinct dams and 495 distinct sires, respectively), and has a maximum depth of nine generations. This structure has been resolved from field observations of maternity and microsatellite-based paternity assignment using the maximum-likelihood method implemented in CERVUS [ 15, 30]. Putative paternal identities were accepted if assigned at a pedigree-wide confidence level ≥80% with a maximum of one allelic incompatibility between sire and offspring. Birthweights were available for 2,902 individuals born between 1985 and 2005 (though no data were collected in 2001 owing to the UK outbreak of foot and mouth disease). For animal model analyses, this was reduced to 2,630 individuals with known mothers.

### Animal model analysis.

Quantitative genetic parameters were estimated using animal models to partition variance into genetic and environmental components. Animal models are able to accommodate unbalanced datasets and complex pedigrees typical of natural populations [ 21], and also allow inclusion of fixed effects to account for known influences on the phenotypic mean. Here we included sex, litter size (twin versus singleton), and maternal age, which are all known to influence birthweight in Soay sheep. Year was additionally included (fitted as a factor) to remove the direct effect of environment on the phenotypic mean (as opposed to effects on variance components). Random effects were then included to model a maternal permanent environment effect (c *_j_*), as well as additive (a *_i_*) and maternal genetic (m *_j_*) effects on birthweight. This random-effects structure results in the total phenotypic variance ( *V*_P_) being split into four components: additive genetic variance ( *V*_A_), maternal genetic variance ( *V*_M_), maternal permanent environment variance ( *V*_C_), and the residual (or temporary environment) variance ( *V*_R_). Importantly, rather than using a conventional animal model, we fitted the maternal genetic effect m *_j_* as a polynomial function of environmental quality *E* using random regression. Here, environmental quality *E* is defined as the proportion of lambs surviving until October 1 in the year of birth (standardized to the interval −1 ≤ *E* ≤ 1). Thus, the birthweight of any animal *i* having mother *j* is given as:



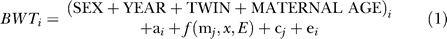



Where *BWT* is birthweight, e *_i_* is a residual error term (having mean zero and variance *V*_R_), and *f(*m *_j_,x,E)* is the random-regression function of maternal genetic value on orthoganol (Legendre) polynomials of *E* with order *x*. The model structure was fitted using different orders of the polynomial function (0 ≤ *x* ≤ 3) using restricted maximum likelihood implemented in ASReml (VSN International; http://www.vsn-intl.com). These alternative formulations of the model were then compared statistically using likelihood ratio tests. Model convergence was not achieved for *x* > 3 (unpublished data). As in a conventional animal model, an implicit assumption here is that *V*_A_ and *V*_R_ are constant across environments. Univariate animal models in data subsets corresponding to good and bad environments (based on upper and lower 50 percentiles of *E*) provided support for this assumption, with no significant differences in estimated additive or residual variances.

The estimated variance–covariance matrix of random-regression parameters for the maternal genetic effect (matrix **Q**) was used to determine the maternal genetic variance–covariance matrix ( **M**) as:



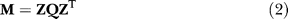



Where **Z** is the vector of orthogonal polynomials evaluated at observed values of *E* for −1 ≤ *E* ≤ 1 (and **Z** ^T^ is the transpose of **Z**). Approximate standard errors for each element of **M** were determined following Fischer et al. [ 11]. The total heritability was evaluated as *h* ^2^_T_ = ( *V*_A_ + *V*_M_/2)/ *V*_P_ (assuming zero covariance between additive and maternal genetic effects [ 22]). Note that *V*_P_ was determined as the sum of estimated variance components and therefore excludes variance attributable to the fixed effects fitted in the model. Finally, **M** was rescaled to a maternal genetic correlation matrix.

### Selection analysis.

Neonatal survival was used as the fitness component *(W)* and was defined as 0 for animals that died before October 1 in the year of birth and as 1 for those that survived. Note that since offspring share common mothers, individual birthweights cannot be considered as independent data points. To account for this non-independence, we used a linear mixed model (with a binomial error structure), with maternal identity fitted as a random effect such that:



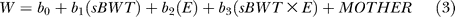



where *E* is environmental quality; *sBWT* is birthweight standardized to mean 0 and variance 1, *b*_0_ to *b*_3_ are constants, and *MOTHER* is the maternal identity. As *E* is determined from the average neonatal survival in each year, a statistical association between fitness and environment is inevitable (since by definition more individuals will have *W* = 1 when *E* is higher). However, it is the effect of *E* on selection, assessed from *b*_3_, the interaction between *sBWT* and *E*, that is of primary interest. Selection differentials *(S)* and standardized selection gradients (β) [ 24] were determined as measures of the strength of selection on *BWT.* Annual phenotypic responses *(R)* to selection were determined as the product of *h* ^2^_T_ and *S,* and the weighted geometric mean response was calculated. Annual responses were weighted by surviving cohort size on October 1 (since, all else being equal, larger cohorts will make greater contributions to future phenotypic distributions).
